# Bats and Viruses: Emergence of Novel Lyssaviruses and Association of Bats with Viral Zoonoses in the EU

**DOI:** 10.3390/tropicalmed4010031

**Published:** 2019-02-07

**Authors:** Rebecca Shipley, Edward Wright, David Selden, Guanghui Wu, James Aegerter, Anthony R Fooks, Ashley C Banyard

**Affiliations:** 1 Wildlife Zoonoses and Vector-Borne Diseases Research Group, Animal and Plant Health Agency (APHA), Weybridge-London KT15 3NB, UK; Rebecca.Shipley@apha.gov.uk (R.S.); david.selden@apha.gov.uk (D.S.); guanghui.wu@apha.gov.uk (G.W.); tony.fooks@apha.gov.uk (A.R.F.); 2 School of Life Sciences, University of Sussex, Falmer, Brighton BN1 9QG, UK; ew323@sussex.ac.uk; 3 APHA—National Wildlife Management Centre, Wildlife Epidemiology and Modelling, Sand Hutton, York YO41 1LZ, UK; james.aegerter@apha.gov.uk; 4 Institute for Infection and Immunity, St. George’s Hospital Medical School, University of London, London SW17 0RE, UK; 5 Department of Clinical Infection, Microbiology and Immunology, Institute of Infection and Global Health, University of Liverpool, Liverpool L69 7BE, UK

**Keywords:** rabies, lyssavirus, bats, emerging, novel, zoonoses

## Abstract

Bats in the EU have been associated with several zoonotic viral pathogens of significance to both human and animal health. Virus discovery continues to expand the existing understating of virus classification, and the increased interest in bats globally as reservoirs or carriers of zoonotic agents has fuelled the continued detection and characterisation of new lyssaviruses and other viral zoonoses. Although the transmission of lyssaviruses from bat species to humans or terrestrial species appears rare, interest in these viruses remains, through their ability to cause the invariably fatal encephalitis—rabies. The association of bats with other viral zoonoses is also of great interest. Much of the EU is free of terrestrial rabies, but several bat species harbor lyssaviruses that remain a risk to human and animal health. Whilst the rabies virus is the main cause of rabies globally, novel related viruses continue to be discovered, predominantly in bat populations, that are of interest purely through their classification within the lyssavirus genus alongside the rabies virus. Although the rabies virus is principally transmitted from the bite of infected dogs, these related lyssaviruses are primarily transmitted to humans and terrestrial carnivores by bats. Even though reports of zoonotic viruses from bats within the EU are rare, to protect human and animal health, it is important characterise novel bat viruses for several reasons, namely: (i) to investigate the mechanisms for the maintenance, potential routes of transmission, and resulting clinical signs, if any, in their natural hosts; (ii) to investigate the ability of existing vaccines, where available, to protect against these viruses; (iii) to evaluate the potential for spill over and onward transmission of viral pathogens in novel terrestrial hosts. This review is an update on the current situation regarding zoonotic virus discovery within bats in the EU, and provides details of potential future mechanisms to control the threat from these deadly pathogens.

## 1. Introduction

The global discovery of lyssaviruses is of continued scientific interest and is of importance to both public and animal health. Lyssaviruses are known to cause fatal encephalitis, referred to as rabies. The term rabies has induced terror throughout human history, as the rabies virus (RABV) is the only viral pathogen that is associated with 100% fatality following the onset of the clinical disease [1]. Whilst rabies is predominantly circulating within domestic and feral dog populations globally, the presence of lyssaviruses in bats is well established [2]. Historically, rabies’ association with hematophagous bats (Desmodus sp., although primarily *Desmodus rotundus*) across the Caribbean, and Central and South America, has both embedded a fear of rabies into human populations, as well as driven an irrational and unjustified fear of bats across many cultures. Certainly, bat transmitted human RABV is rare, although in areas where terrestrial rabies has been eliminated, bat rabies remains a constant threat, as exemplified by continued human cases of bat rabies across North America [3]. In endemic areas, human infection with dog rabies results in thousands of human deaths annually. The estimates of human infection are thought to be conservative, because of inadequate diagnostic and reporting systems across Africa and Asia [4]. Wildlife species can also play an important role in the epidemiology of disease, although the paucity of data on wild animal populations, their distribution, and the generally sporadic interactions between different wildlife populations and domesticated carnivore species means that the role of wildlife and the epidemiology of the virus is often unclear. Still, the transmission of the virus between wildlife and domestic terrestrial carnivores is multidirectional, with incursions of domestic dog rabies into fox populations being reported [5].

The severity of disease caused by lyssaviruses means that the potential for cross species transmission events (CSTs) is of significance to human and animal populations [6]. For the rabies virus, spill over events are considered as those that result in dead-end infection, whilst CSTs result in the sustained onward transmission of the virus in the new host. Spill over from bats species appears common for RABV in the Americas, whilst events involving the other lyssaviruses across the Old World appear to be rare. Whilst spill over events for lyssaviruses have been reported, host switching events are far rarer and have only been described for RABV in the Americas [5,7,8,9]. The factors involved in CSTs with the sustained onward transmission of the virus remain undefined, and endeavours to identify specific amino acid substitutions facilitating virus adaptation to new host species have been, on the most part, unsuccessful. Kuzmin et al. (2012) observed that for sustainability within a bat population, a Serine at position 242 in the viral G protein appeared to predominate, and that contrastingly, an Alanine/Threonine substitution at position 242 appears to facilitate RABV sustainability within the carnivore population [10,11]. Intensive characterisation of the genetics within viral populations, including quasispecies, may elucidate the molecular mechanisms that facilitate lyssavirus adaption, however opportunities to genetically characterise such events are rare. 

## 2. The Increasing Diversity of the Bat Lyssaviruses

**Figure 1:**
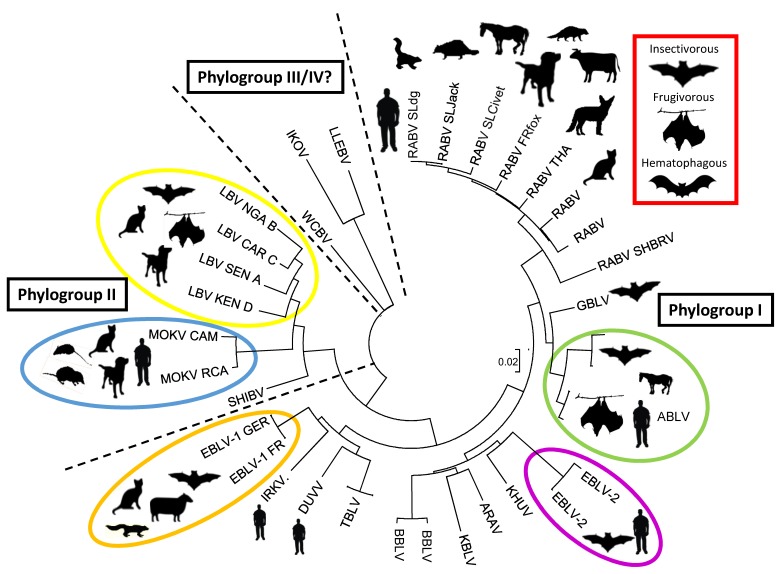
Phylogenetic relationships of the lyssaviruses. The phylogenetic tree is based on an alignment of a fragment of the lyssavirus nucleocapsid gene (450bp). The evolutionary history was inferred using the neighbor-joining method, with branch lengths in the same units as those of the evolutionary distances used to infer the phylogenetic tree. Evolutionary analyses were conducted using MEGA6.

Alongside RABV, which is both associated with the infection of terrestrial carnivore species and the chiroptera, fifteen other genetically-, and to some extent, antigenically-related viruses exist within the lyssavirus genus (Figure 1) [12]. Of these, 13 have been isolated from bat species, with the Mokola lyssavirus (MOKV) and Ikoma lyssavirus (IKOV) being the only two viruses that have no current association with bat species (Table 1). Whilst MOKV has been isolated on numerous occasions from rodent species [13,14], IKOV exists only as a single isolate from a rabid African civet (*Civetticus civetticus*) [15]. Enhanced surveillance activities are required in order to understand these isolations, and may not only inform on the natural reservoir host for these lyssaviruses, they may also facilitate the discovery and isolation of novel lyssaviruses from different hosts. A recently identified virus from Asia, named Taiwan Bat Lyssavirus (TBLV), is tentatively associated with the lyssavirus genus. Two separate isolations of TBLV have been reported from the Japanese house bat (*Pipistrellus abramus*) [16]. 

Whilst the epidemiology of RABV is well defined, being present in terrestrial carnivores globally and bat species within the New World, the epidemiology of the other lyssaviruses is poorly understood, with only single isolates being available for several species (Table 1). However, from an epidemiological standpoint, other than RABV, all other lyssaviruses appear absent from the New World, being described solely within terrestrial or bat species across the Old World. Regardless, the paucity of the epidemiological data for the lyssaviruses may reflect fewer cases of infection with these viruses than there are with RABV across human and animal populations, or, conversely, may be due to the inability of the existing diagnostic procedures used in endemic areas to differentiate between lyssavirus species. The fluorescent antibody test (FAT) is the most common diagnostic tool used for antigen detection, however it is unable to differentiate between the lyssavirus species. Laboratories in endemic areas do not generally have the capability, often through limitations in resources, to perform secondary confirmatory testing, such as PCR, and sequencing, so as to genetically type the virus to identify which lyssavirus is present in FAT positive samples [17]. The recent adoption of molecular tools for lyssavirus diagnosis by the World Organisation for Animal Health (OIE), will help to overcome this obstacle to virus identification; as more divergent lyssaviruses are discovered, the ability of commercial conjugates to detect them needs constant re-evaluation. Molecular differentiation will resolve the epidemiological status of each virus, and consequently, will help to understand the threats of each lyssavirus to animal and human populations [18]. 

Lyssaviruses in Europe were first reported in 1954 in Germany [19]. In 1955, a lyssavirus was isolated from insectivorous bats (*Nyctalus noctula*) from the FR Yugoslavia, which confirmed bat rabies [20]. Highly divergent lyssaviruses that reacted differently to monoclonal antibody panels when typed were initially discovered in 1956, originating with the description of the Lagos Bat virus in Africa. Prior to the advent of molecular testing, serological profiling using monoclonal antibodies was utilised to distinguish between lyssavirus species, and revealed virus isolates that were capable of causing rabies, but that reacted differently to defined panels of monoclonal antibodies [21]. The advancement of molecular methods, such as PCR and sequencing technologies, have superseded the antibody-based classification of new pathogens [22]. PCR and sequencing allow for the immediate genetic analysis of the suspect material, and their application to suspect material has led to the rapid typing of numerous novel lyssaviruses, initially often through genetic typing [23,24,25,26]. Although the true burden of novel lyssaviruses remains undefined, the potential for fatal infection following spill over events highlights the importance of the characterisation and classification of all lyssaviruses. The discovery of novel lyssaviruses has warranted a heightened interest in bats. As defined reservoirs of many zoonotic pathogens, the viruses harbored by bats are capable of causing explosive outbreaks of disease in human or animal populations following a cross species transmission event. In some areas, these transmission events have increased proportionally to the increased intrusion of human populations into areas of bat habitation [27], as well as the increasing popularity of leisure activities and occupations that involve entering habitats frequented by bats (e.g., caving and potholing).

The lyssavirus species have a distinct and unique epidemiology through their association with bats [28]. Classical RABV is present globally, being reported in terrestrial carnivores, herbivores, and across the New World within multiple bat species. Whilst terrestrial rabies has been largely eliminated in the Americas, it is still associated with the infection of insectivorous; hematophagous; and, to a lesser extent, frugivorous bats. Interestingly, of the 16 classified lyssaviruses, only classical RABV has been reported in the Americas, and the current bat population represents an omnipresent source of RABV infection, for which elimination options are very limited. Certainly, the potential for host switching events to occur into both animal and human populations persists with any resulting human fatalities being reported. In contrast to the situation across the Americas, classical RABV has never been detected in bats in the Old World [29], yet it exists in terrestrial carnivore populations globally. From a bat infection perspective, a further contrasting feature of RABV infection is the association with different bat hosts. Bat rabies in the New World has been detected in over 40 different bat species, although infection is most typically associated with a handful of chiropteran species. In contrast, the Old-World lyssaviruses appear to be most commonly associated with a single or restricted host bat reservoir species. For example, EBLV-1 is predominantly associated with *Eptesicus serotinus*; EBLV-2 with *Myotis daubentoniid*, and so on. This species specific detection virus–host relationship has led to the suggestion of host restriction or co-evolution of pathogens with certain bat species, although evidence for either is scant. Further occasional cases of presumed spill over infection are reported in other species, although this appears to be rare (Table 2). EBLV-1 has been reported in sheep, cats, and a stone marten, although onward transmission within the new host has not been demonstrated. With several bat lyssavirus species, the detection of only a handful of cases of each isolate, or in some cases, only a single isolation, precludes a thorough and accurate assessment of the viral epidemiology. The basis for the apparent abundance of bat lyssaviruses in the Old World, but only bat RABV in the New World, remains an enigma. 

**Figure 2:**
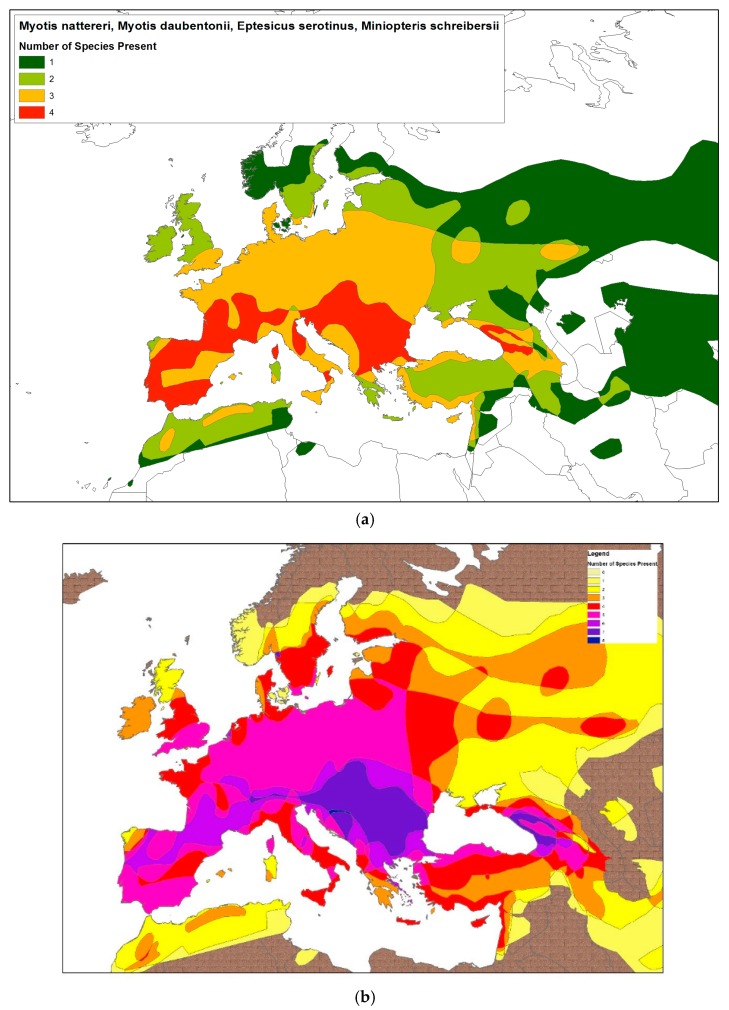
(**a**) Risk map for bat species most commonly reported as infected with lyssaviruses in the EU. Data includes ranges of *Eptesicus serotinus* (EBLV-1), *Myotis daubentonii* (EBLV-2), *Myotis nattereri* (BBLV), and *Miniopteris schreibersii* (LLEBV and WCBV). Data derived from IUCN (https://www.iucn.org/). (**b**) Risk map for all bat species associated with lyssavirus infection across Europe. Data includes ranges of *Eptesicus serotinus* (EBLV-1), *Myotis daubentonii* (EBLV-2), *Myotis nattereri* (BBLV) and *Miniopteris schreibersii* (LLEBV and WCBV), *Myotis mystacinus* (KHUV), *Myotis brandtii* (KBLV), *Myotis blythii* (ARAV), and *Murina leukogaster* (IRKV).

From a risk perspective, the known host ranges for lyssaviruses give an indication as to the areas where bat species can be found, and hence, a risk, albeit low, of human–bat interaction must exist. For the most commonly detected lyssaviruses, this range extends across much of the European Union (Figure 2). However, to date, only two human infections with bat lyssaviruses have been described within Europe, both involving fatalities associated with EBLV-2 (Table 2) [30,31,32]. 

## 3. The Association of Other Viral Zoonoses with European Bat Species

Published epidemiological studies have associated viral zoonoses with 45 different species of bat within European countries, which cluster within 5 families [60]. Of these 45 species, the majority (37) sit within the *Vespertilionidae*, which includes *Myotis, Eptesicus*, *Pipistrellus* and *Plecotus*. The *Vespertilionidae* is not only the largest family of bats in Europe but also the most geographically dispersed (Table 3). Some Vespertilionids are found throughout Europe, for example *Myotis nattereri* and *Pipistrellus pipistrellus*, while others have restricted ranges, such as *Myotis punicus* and *Plecotus sardus*. Four other chiropteran families are represented in Europe, *Rhinolophidae, Miniopteridae, Molossidae* and *Pteropodidae*, with just single species representing the latter three families. Finally, all but one species recorded in Europe are insectivorous, with the Egyptian fruit bat (*Rousettus aegyptiacus*) as a frugivorous exception, found in Cyprus and southern Turkey [61,62].

The diversity observed in bat species across Europe also extends to the range of viruses that they have been shown to harbour. Many species have been reported antigen or antibody positive for lyssaviruses (Table 1). However the association of bats with other zoonoses is also of interest. The 2014 outbreak of Ebola in West Africa highlights the potential for bat pathogens to spill over into human populations. It is generally accepted that this outbreak initiated through human-bat interaction, although some controversy surrounds this conclusion [116]. Regardless, the threat of bat viruses crossing the species barrier and entering human populations exists and must be considered a significant threat to public health in areas where bats and humans live in close proximity. Alongside the risk from lyssaviruses several other viruses of note have been identified in European bat species. Filoviruses represent one of the most feared viral families from the perspective of human health as infection has been sensationalised through films and books and explosive outbreaks have been recorded although the outbreaks themselves have often been self-limiting. Filoviruses can cause a lethal hemorrhagic fever in humans and nonhuman primates and the association with bats and other potential reservoir hosts remains undefined [117]. As with the lyssaviruses, the filovirus family is continually expanding with the discovery of novel isolates in different parts of the world [118]. Although primarily associated with primate infection, several bat species have been associated with filoviruses; with both the ebolavirus and marburgvirus genera reported as being associated with different bat species. For the ebolavirus genus the association is predominantly through the large fruit bat species (*Hypsignathus monstrous, Epomops franqueti and Myonycteris torquata*) whilst Marburgvirus infection has been linked to both fruit bats (*Rousettus aegyptiacus*) and insectivorous bats (*Rhinolophus eloquens, Miniopterus inflatus and Miniopteris schreibersii*). The association of the latter species in Spain and Hungary with a novel ebolavirus, Lloviu virus (LLOV), led to a heightened awareness of bats as potential reservoirs of zoonoses [119]. Large insectivorous bat die off events have been reported historically in colonies of Schreiber’s bats (*Miniopterus schreibersii*) in Spain, France and Portugal [120]. In Spain, these mortality events prompted investigation into the potential cause of the mortalities and although no causal relationship between filoviruses and the die off were established, the detection of LLOV in *Miniopteris schreibersii* is of potential concern to human and animal health. As described above, this species of bat has also been associated with the highly divergent Lleida bat lyssavirus and as such represents an important possible threat to human health as vaccines or antivirals specific for either infection do not exist; therefore, the consequences of any infection would be very serious. A further concern of filovirus infection of bats is the apparently asymptomatic nature of natural infection, which makes it difficult to identify the infected bats. Whilst bats are considered to be the reservoir host for lyssaviruses, infection will often result in the development of clinical disease and death. Lyssavirus natural infection is hard to define in terms of disease presentation but where experimental studies have been performed in bats, lyssavirus infection typically results in the development of rabies where intracranial inoculation is concerned but with either the development of disease or survivorship and seroconversion following inoculation by peripheral routes. Contrastingly, numerous experimental studies have demonstrated that bats experimentally infected with filoviruses remain healthy post infection but can shed viral products in fecal matter for several weeks [115,121,122]. Interestingly, LLOV was only detected in association with diseased bats with no virus, or vial products, being associated with healthy Schreiber’s bats or co-roosting species. This is considered unusual as filovirus infection of other bat species is generally asymptomatic, so the isolation of virus from only diseased bats may indicate differences in pathogenicity for LLOV compared to other filovirus infections of bat. Certainly, the relationship between filovirus and bats requires further investigation as these viral pathogens pose potential threats to humans where interactions with bats occurs. 

As well as lyssa- and filoviruses, bats within the EU have been also linked with coronavirus infection. Severe Acute Respiratory Syndrome Coronavirus (SARS-CoV) and Middle East Respiratory Syndrome Coronavirus (MERS-CoV) are coronaviruses capable of infecting humans resulting in a clinical disease of respiratory and gastrointestinal systems [123]. In animal reservoirs, coronavirus infection can result in respiratory, neurological or hepatic disease [124]. Since 2003, numerous novel coronavirus species have been isolated. Within the Alphacoronavirus and Betacoronavirus genera, nine of the 18 recognised viral species have been identified in bats [125]. Moreover, two high profile human disease outbreaks, Severe Acute Respiratory Syndrome (SARS) and Middle East Respiratory Syndrome (MERS) have been phylogenetically linked to a zoonotic viral origin in bats [126,127]. Most coronaviruses are associated with insectivorous bats (*Rhinolophus sinicus, Rhinolophus macrotis, Rhinolophus ferrumequinum, Chaerephon plicata, Rhinolophus pusillus, Rhinolophus blasii, Tylonycteris pachypus, Pipistrellus abramus, Neoromica capensis, Vespertilio superans*) [126]. Bat-to-human transmission of coronaviruses is likely very rare, if it occurs at all, and it is more common for bats to infect another terrestrial animal which subsequently infects humans. After the discovery and characterisation of SARS-CoV circulating in masked palm civets sold at Chinese markets and then later in horseshoe bat populations (*Rhinolophus*) in China, it was suggested that bats were the primary reservoir and the civet infection was a result of a spill over event [128,129,130,131,132]. In addition to this, the initial detection of MERS-CoV in dromedary camels in Saudi Arabia suggested that camelids may be a reservoir host for this pathogen but subsequent studies have reported a single isolation from fecal matter from a *Taphozous perforates* bat and a β-coronavirus, with 96.5% amino acid identity to the MERS-CoV, has been isolated from a *Nyctinomops laticaudatus* bat [127,133]. This warrants the speculation that either the MERS-CoV reservoir resides in a bat species and remains to be discovered, or that dromedary camels are the primary reservoir for MERS-CoV that originated following cross species transmission of a viral ancestor that once resided in a bat populations. The coronvirus surface-located trimeric spike glycoprotein (S) dictates the coronavirus host range as it specifically binds certain receptors for infection [134,135] and so studies surrounding receptor utilisation are warranted in determining any host restriction for these viruses. Rhinolophid bats in China have been described as hosts to many SARS-like coronaviruses, some of which are proposed to be the direct ancestors of SARS-CoV [136]. Each of these pathogens has the potential to cross the species barrier and cause disease outbreaks in terrestrial species, a process though to be driven by adaptation to the new host through genetic mutation [137]. Whilst many of the exact mechanisms required for a spill over event to occur are largely undefined, the S protein and host receptors are the logical starting point and key binding sites of the S protein and potential host receptors remain to be characterised for multiple pathogens. Certainly, identification of receptor binding domains may reveal host tropism patterns and enable evaluation of virus spill over potential.

Further to this, many other potentially highly pathogenic viruses, such as paramyxoviruses, bunyaviruses and hantaviruses, have been detected in European bats (Table 3). While similar viruses to these have previously been isolated across the globe and have shown to be a high risk to animal and human health, the precise risk posed by these novel viruses found in European bats is unknown. This is because for the majority of these viruses, detection is limited to nucleic acid or antibody detection with very little laboratory analysis having been performed.

## 4. Evolution of Viral Species within Bats

Surveillance programmes for bat lyssaviruses commonly report a diverse range of wild bat species apparently participating in the epizootiology of lyssaviruses. Spill over between ecologically distinct species appears to be common, with the divergence dates for viral clades often measured as evolutionarily recent events (i.e., <1000 years) [88,138,139,140]. The sheer diversity of bat species associated as potential reservoir hosts to different lyssavirus species suggests historic spill over events and the subsequent maintenance of disease in novel hosts. This would have ultimately promoted new disease reservoirs and the speciation of lyssaviruses. From a host perspective, factors such as the fundamental ecology of roost choice by individual temperate bats, which may fall within geographical locations consistent with what is now defined as the EU, have mediated intraspecific and interspecific transmission events. 

The potential for virus transmission between bats requires close contact with lyssavirus transmission, likely requiring physical and direct contact, such as biting and scratching, in the absence of efficient aerosol spread. Away from their roosts, most temperate European species (i.e., insectivores in seasonal biomes) spend the majority of their time foraging and in flight. Most species appear to forage alone, and when foraging bats meet, interaction appears limited to chasing behavior and social calling. If in-flight contact does occur, significant injuries to delicate wings would probably be fatal. Additionally, the evidence suggests that lyssavirus excretion (and hence bat infectivity) is usually associated with the final symptomatic stages of disease, and is often accompanied by ataxia and a flightless or moribund state, further reducing the likelihood that transmission occurs between bats in flight or in those that are away from roosts. The transmission of lyssaviruses within roosts therefore seems most likely, and with is what is known for the transmission of other viral pathogens, interactions within the roost structures would seem to be most favorable for disease transmission.

Historically, at the end of the last glacial maxima, the range of most European species is considered to have been constrained to multiple refugia around the Mediterranean basin and across to the Caucasus. At this time, northern Eurasia would presumably have been inhospitable to all bats. Both cave hibernator and tree hibernator bats (both classes of bats comprising species that use crevices and cavities for their nursery roosts) would have been competing for the same resource in the summer, across the same constrained landscape, so that direct interspecific contact, whilst infrequent, may have exceeded that which is documented today. In winter, the seasonal migration of cave hibernators would have resulted in interactions with cave specialists (a third class of bats that use caves for both nursery roosts and hibernation) to produce the substantial aggregations of many species. Today, tree hibernating bats will also periodically use caves (for either nursery sites or hibernacula in southern European countries), and it seems likely that this also occurred in the past. Thus, the whole European bat fauna may have frequently shared key sites in winter across the Mediterranean and Pannonian basins, as well as the Caucuses. Ancestral pathogens circulating in this environment would have benefited from the size and longevity of the colonies interacting in the most suitable caves, as well as the relatively frequent and prolonged opportunities for heterospecific transmission. However, an environment such as this would favor the most transmissible viruses, and these consequently would have become the most dominant/prevalent.

As the post glacial climate warmed across Europe, forested landscapes are thought to have extended northward, especially towards those forest communities of deciduous trees producing a diverse and abundant supply of cavities and crevices suitable for nursery sites for cave hibernating and tree hibernating species. This would have substantially extended the summer ranges of the tree roosting species, and whole populations of cave hibernators may have become disconnected from caves in their Mediterranean refugia, breeding in northern forests and hibernating in northern caves nearby. Cave specialists (including *M. myotis* and most *Rhinolophidae*) presumably remained fixed in their previous ranges, restricted to a range similar to that held today by other cave specialists such as *M. blythii, M. capaccinii*, and *Miniopterus schreibersii.* These changes in the abundance and distribution of tree roosting species may have driven opportunities for the diverse speciation of lyssaviruses across Europe, as distinct and discrete populations of some species emerged from their glacial refugia. Concomitantly, the dynamics of the cave specialists are proposed to have remained largely unchanged, and, as such, these cave dwelling species may have continued to circulate a common pool of ancestral lyssaviruses as well as other pathogens. 

Finally, the present-day environment has been radically expanded and diversified by man to provide roost resources to all European bats. Mines and buildings have enriched the resources available to tree roosting species, as well as extending the geographical range of cave specialists. For the many tree roosting species that use buildings in the summer, it is not clear if the loss in the quantity of tree roosts following the reduction in the forested area is compensated for by their provision in buildings. For cave specialists, such as *M. myotis* and some *Rhinolophidae*, their exploitation of anthropogenic sites (buildings in summer, and local mines or caves in winter) has presumably led to a considerable northward extension of their ranges. As well as extending the sympatry of these expansive cave specialists with many tree roosting species, this process will have fragmented their populations, producing independent northern European populations, disconnected from their Mediterranean peers.

Buildings not only encourage heterospecific co-roosting in summer, when activity is much greater, but also facilitate “super roosts”, by presenting unnatural combinations of environmental qualities, such as size, as well as, occasionally, additional anthropogenic heat, enabling unnaturally large aggregations of many tree roosting species that would be impossible in natural sites. Virus epizootiology would theoretically benefit from these increased opportunities for intra-specific and inter-specific transmission. 

Lyssaviruses circulating in modern European bats may now have many new ways to spread and evolve, driven by the changes to the dynamics and interactions of their bat hosts. Cave specialists, and the viruses they have independently maintained in their perpetually large and aggregated Mediterranean populations, will have presumably been carried northward across substantially extended ranges, to mix with a diverse community of tree roosting species. As well as reacquainting populations of northern European tree roosting species with viruses they may not have seen for over 5000 years, this process also permits any viral pathogen that has co-evolved in a tree roosting species to move the other way into the more densely aggregated populations of cave specialists, although evidence of bat host switching is scant. Buildings, and the diversity of roost options offered, are the primary conduit for this potential interchange, bringing otherwise ecologically separate populations into close contact. Certainly, if the fragmentation of host bat populations promoted a sufficient evolutionary drift to produce antigenically distinct viruses, it can be imagined that this might lead to a substantial proliferation of disease, and perpetuate its spread to many new bat species. Antigenically similar viruses, even if they have co-evolved in separate cave hibernating species, may compete more directly for the new pool of hosts.

## 5. Bats and Their Role as a Reservoir for Viral Pathogens

Different chiropteran species are widely accepted as reservoir hosts for lyssaviruses and other viral pathogens, as described above. However, for the lyssaviruses, this interaction is poorly understood. Indeed, lyssaviruses and bats do not typically exhibit the classical symbiotic relationship expected of co-evolution, as the lyssavirus infection of bats is most commonly detected following the observation of clinical disease. This contrasts with other viral pathogens, for which bats are considered natural reservoirs, including filo- and henipa-viruses. Both the natural and experimental infection of bats with these other pathogens results in the shedding of the virus, often in the complete absence of clinical disease. Furthermore, evidence has shown that bats, and other mammals, are able to mount a sufficient immunological response following viral exposure, and successfully manage to clear the virus before the onset of clinical disease. For lyssaviruses, the repeated detection of healthy seropositive bats in different roost populations strongly suggests that exposure events can result in viral clearance, following the development of a localised and/or systemic immune response. Alongside this, recent studies have described serologically positivity in unvaccinated humans within Amazonian populations [141]. Again, this suggests that lyssavirus exposure can result in clearance, most likely where detected, through the development of a humoral response. What drives this human exposure to RABV, however, is less clear with hunting of bats, as well as the feeding of vampire bats on humans being plausible exposure opportunities. Certainly, the structure of Amazonian populations, and the overlap in bat and human habitations, may facilitate the exposure to a lyssavirus, whether it be via bite or non-bite transmission. Also, the mechanisms involved in clinical disease manifestation following natural infection remain ill-defined, so the outcome of lyssavirus infection/exposure in bats versus another mammal cannot yet be accurately compared. It may be so that bats exhibit much longer incubation periods than other mammals, facilitating in the maintenance of the virus in bat populations. However, the lack of knowledge surrounding, specifically, the innate signalling mechanisms in bats following exposure, prohibits any understanding surrounding their status as the lyssavirus reservoir host. Related to this is the observation of clinical disease in a U.K. Daubenton’s bat nine months following captive care [142]. This, again, demonstrates that these viruses can exist within bat species for long periods of time, before the development of clinical disease. How the virus is maintained and where the virus hides during prolonged periods post infection before the development of disease remains unknown. 

## 6. Availability of Human Vaccines for Bat Pathogens

For lyssaviruses, the lack of vaccine protection against numerous divergent lyssaviruses has been previously defined. However, the continued emergence of novel lyssaviruses warrants the continued assessment of vaccine protection, as well as where human infection occurs and how the clinical disease develops, as there remains no cure for rabies. Human rabies vaccines have been available for decades, and following vaccination, it has long been established that a neutralising antibody titre over a defined threshold will protect individuals from the development of disease when infection with classical RABV occurs [143]. However, the protective cut-off for a serological neutralising antibody titre is poorly defined for many of the other lyssaviruses within the genus, and consequently, the discovery of novel viruses warrants an investigation on the efficacy of existing pre- and post-exposure preparations [144,145,146,147,148,149,150]. Although response to vaccines differs between individuals, it is widely accepted that a protective titre of 0.5 international units (IU)/ml is sufficient for protection against RABV [151]. Alongside RABV, the current vaccine protects against all phylogroup I lyssaviruses, namely: ARAV, ABLV, BBLV, DUVV, EBLV-1, EBLV-2, IRKV, KHUV, and GBLV, although the level of neutralising antibody required to protect is undefined. Evidence has shown, however, that a titre higher than 0.5IU/ml is required for protection for some phylogroup I lyssaviruses, indicating an increased antigenic distance of the vaccine strains to the circulating lyssaviruses [144] [146,149]. For more divergent lyssaviruses, such as those in phylogroup II and III, in vivo vaccination-challenge experiments have shown that the antibody response generated from the RABV vaccine is not sufficient for protection [17,147,150,152,153,154]. 

For other viral zoonoses of bat present within the EU there have been varying degrees of success in the development of vaccines. Following the 2014 Ebola outbreak, a vaccine based on vesicular stomatitis virus (VSV) expressing the surface glycoprotein of Zaire Ebola (EBOV), termed VSV-EBOV, was developed [155]. Clinical trials demonstrated 95-100% efficacy in generating a protective response against EBOV, making it the first filovirus vaccine in use [156]. However, as with the lyssaviruses, there is antigenic divergence across the filovirus family with six distinct species of ebolavirus being described and little is understood regarding any potential cross-protection afforded by the current vaccine. Further, two other genera are classified alongside the ebolaviruses within the filovirus family, Marburgviruses and Cuevavirus. Whilst a vaccine for Marburgviruses is not currently in use, studies have shown that, similar to EBOV, VSV expressing MARV glycoproteins is effective at generating a sufficient antibody titre for protection in non-human primates [157]. Of note, the VSV-MARV vaccine affords protection against Ravn virus (RAVV), a further novel lyssavirus [158]. 

The only currently available vaccine for coronaviruses is a canine vaccine although multiple studies are focused on creating vaccines for SARS-CoV and MERS-CoV. All coronavirus vaccine candidates are directed against the Spike protein (S protein), the most immunological component of coronaviruses. Both DNA vaccines and subunit viral vectored vaccines, such as Adenovirus, Venezuelan equine encephalitis virus and modified Vaccinia virus Ankara expressing the S protein, have been demonstrated provide a sufficient protective neutralising antibody response against MERS-CoV in a murine model [159]. Of note, the route of administration was a key determinant in the scale of the resulting antibody response with intranasal administration being the most effective method for both SARS-CoV and MERS-CoV protection as it stimulated significantly higher IgA antibody response than subcutaneous inoculation [160,161]. Clearly, vaccines for zoonotic viral pathogens of bats need further development.

## 7. Conclusions

Numerous viruses exist in European bat species, some of which currently no effective human or animal vaccines are available. Vaccination of bats, through their protected status in the EU is unlikely to ever be a viable option although developments in vaccine applications for chiroptera may have future applications in preventing the disease in wild bat populations [162]. For lyssaviruses, the OIE and WHO have targeted 2030 for the elimination of dog-mediated human rabies. It is possible that should this be achieved, the spill over of bat lyssaviruses may become more evident and host switching events may occur with pathogens for which there is no vaccine protection. Certainly, the recent detection of highly divergent lyssaviruses (LLEBV) and filoviruses (LLOV) in bats across the EU poses potential the risk to human populations, especially as vaccines or antiviral drugs against these viruses do not exist. Further studies are required to understand: the mechanisms of both maintenance and transmission of viral pathogens within bat populations; the zoonotic potential of viral pathogens detected in bats; and the risk of host switching events that may impact on human and animal health.

## Figures and Tables

**Table 1:** The association of the lyssavirus species with bats.

Lyssavirus Species	Common Bat Name	Bat Species Associated with Lyssavirus Infection	Countries Reporting Lyssavirus in Bats	Vaccine Protection Predicted?
Aravan lyssavirus (ARAV)	Lesser mouse-eared bat	*Myotis blythi*	Kyrgystan	Y
Australian bat lyssavirus (ABLV)	Black flying fox and related sp.	*Pteropus alecto*	Australia	Y
	Yellow-bellied sheath-tailed bat	*Saccolaimus flaviventris*		
Bokeloh bat lyssavirus (BBLV)	Natterer’s bat	*Myotis nattereri*	Germany, France	Y
Duvenhage lyssavirus (DUVV)	Undefined	*Miniopterus sp.*	South Africa, Kenya	Y
	Egyptian slit-faced bat	*Nycteris thebaica*	Zimbabwe	
European bat 1 Lyssavirus (EBLV-1)	Serotine bat	*Eptesicus serotinus*	France, Germany, and Spain	Y
European bat 2 lyssavirus (EBLV-2)	Daubenton’s bat	*Myotis daubentonii*	The Netherlands, Switzerland, UK, France, Germany, Luxembourg, and Finland	Y
Gannoruwa bat lyssavirus (GBLV)	Indian flying fox	*Pteropus medius*	Sri Lanka	Y
Ikoma lyssavirus (IKOV)	N/A	*N/A*	Tanzania	N
Irkut lyssavirus (IRKV)	Greater tube-nosed bat	*Murina leucogaster*	Russian Federation and China	Y
Kotolahti Bat Lyssavirus (KBLV)$	Brandt’s bat	*Myotis brandti*	Finland	Y
Khujand lyssavirus (KHUV)	Whiskered bat	*Myotis mystacinus*	Tajikistan	Y
Lagos bat lyssavirus (LBV)	Straw coloured fruit bat	*Eidolon helvum*	Nigeria, Senegal, Ghana, and Kenya	N
	Egyptian fruit bat	*Rousettus aegyptiacus*	France (ex-Togo or Egypt), and Kenya	
	Dwarf epaulet fruit bat	*Micropteropus pussilus*	Central African Republic	
	Gambian epauletted fruit bat	*Epomorphus giambianus*	Ghana	
	Buettikofer’s epauletted fruit bat	*Epomops buettikoferi*	Ghana	
	Gambian slit-faced bat	*Nycteris gambiensis*	Guinea	
	Wahlberg’s epauletted fruit bat	*Epomorphorus wahlbergi*	South Africa	
Lleida bat lyssavirus (LLEBV)	Common bent-winged bat	*Miniopterus schreibersii*	Spain and France	N
Mokola Lyssavirus (MOKV)	N/A	*N/A*		N
Rabies lyssavirus (RABV)○	Big brown bat	*Eptesicus fuscus*	North and South America	Y
	Mexican/Brazilian free-tail bat	*Tadarida brasiliensis*		
	Silver-haired bat	*Lasionycteris noctivagens*		
	Tri-coloured bat	*Perimyotis subflavus*		
	Vampire bat	*Desmodus rotundus*		
Shimoni bat lyssavirus (SHIBV)	Commerson’s leaf-nosed bat	*Hipposideros commersoni*	Kenya	N
Taiwan bat lyssavirus (TBLV)$	Japanese house bat	*Pipistrellus abramus*	Taiwan	Y
West Caucasian bat lyssavirus (WCBV)	Common bent-winged bat	*Miniopterus schreibersii*	Russian Federation and Kenya^	N

○: Only bat species most commonly associated with rabies virus infection are listed for clarity. $: Awaiting official classification within the lyssavirus genus. ^: Serological evidence alone.

**Table 2:** Documented lyssavirus spill over events.

Continent	Lyssavirus Species	Country	Presumed Host Species	Spill over Species	Reference
Europe	EBLV-1	Germany	*Eptesicus serotinus*	Stone marten ×1	[33]
		Denmark	*Eptesicus serotinus*	Sheep ×4 (1998), 1 × (2002)	[34]
		France	*Eptesicus serotinus*	Cat ×1 (2003), 1 × Cat (2007)	[35]
		Germany	*Eptesicus serotinus*	Myotis myotis	[36]
			*Eptesicus serotinus*	Myotis daubentonii	
			*Eptesicus serotinus*	Nytalus noctula	
			*Eptesicus serotinus*	Pipistrellus pipistrellus	
			*Eptesicus serotinus*	Pipistrellus nathusii	
			*Eptesicus serotinus*	Plecotus auritus	
		Spain	*Eptesicus serotinus*	Myotis nattereri	[37]
			*Eptesicus serotinus*	Minipterus schreibersii	
			*Eptesicus serotinus*	Rhinolophus ferrumequinum	
			*Eptesicus serotinus*	Barbastella barbastellus	
		Russia	*Eptesicus serotinus*	Human	[38]
	EBLV-2	Finland	*Myotis daubentonii*	Human	[30]
		Scotland	*Myotis daubentonii*	Human	[32]
Oceania	ABLV	Australia	*Chalinolobus gouldii*	2× Horses	[39]
			*Saccolaimus flaviventris*	Human	[40]
			*Pteropus spp.*	Human	[41]
			*Pteropus spp.*	Human	[42]
Africa	MOKV	Nigeria	Unknown	Human	[43]
			Unknown	Human	[44]
		Zimbabwe	Unknown	5 cats; 1 dog	[45]
			Unknown	1 cat	[46]
		South Africa	Unknown	Cat ×1 (1970); Cat ×1 (1995)	[47]
			Unknown	cat × 2 (1996), cat ×2 (1997), cat ×1 (1998)	[48]
			Unknown	1 cat	[49]
			Unknown	dog ×1 (2005), cat ×1 (2006), cat ×1 (2008)	[50]
			Unknown	cat ×2 (2012), cat ×1 (2014)	[13]
		Ethiopia	Unknown	1 cat	[51]
	DUVV	South Africa	*Microchiroptera*	Human	[52]
			*Microchiroptera*	Human	[53]
		Kenya	*Microchiroptera*	Human	[54]
	LBV	South Africa	*Epomophorus wahlbergi*	Cat	[55]
			*Epomophorus wahlbergi*	Mongoose	[56]
		Zimbabwe	*Epomophorus wahlbergi*/*Eidolon helvum*	Cat	[57]
		Ethiopia	*Epomophorus wahlbergi*/*Eidolon helvum*	Dog	[51]
Asia	IRKV	Siberia	*Murina leucogaster*	Human	[58]
		China	*Murina leucogaster*	Dog	[59]

**Table 3:** Association of non-lyssavirus zoonoses with bat species in the EU.

Family	Species Common Name	Species Latin Name	Association with Viral Pathogen?	References
Rhinolophidae	Blasius’s horseshoe bat	*Rhinolophus blasii*	Coronaviruses	[63]
	Mediterranean horseshoe bat	*Rhinolophus euryale*	Coronaviruses	[63]
	Greater horseshoe bat	*Rhinolophus ferrumequinum*	Coronaviruses/Gammaherpesvirus/Adenovirus/Papillomaviruses	[37,63,64,65]
	Lesser horseshoe bat	*Rhinolophus hipposideros*	Coronaviruses/Orthoreovirus/Astrovirus	[66,67,68,69]
	Mehely’s horseshoe bat	*Rhinolophus mehelyi*	Coronavirus	[63]
Vespertilionidae	Western Barbastelle bat	*Barbastella barbastellus*	Carmovirus	[70]
	Isabelline Serotine bat	*Eptesicus isabellinus*	Coronavirus	[71,72]
	Common Serotine	*Eptesicus serotinus*	Coronavirus/Herpesvirus/Papillomavirus	[36,73,74,75,76,77]
	Savi’s pipistrelle	*Hypsugo savii*	Coronavirus	[67,78,79]
	Alcathoe whiskered bat	*Myotis alcathoe*	Paramyxovirus	[80]
	Bechstein’s bat	*Myotis bechsteinii*	Astrovirus/Coronavirus/Paramyxovirus	[81,82,83]
	Lesser mouse-eared bat	*Myotis blythii*	Coronavirus	[72,75,84]
	Long-fingered bat	*Myotis capaccinii*	Paramyxovirus	[80,85]
	Pond bat	*Myotis dasycneme*	Coronavirus	[83,86,87,88]
	Daubenton’s bat	*Myotis daubentonii*	Atrovirus/Coronavirus/Paramyxovirus	[63,80,81,83,85,87,89,90,91,92]
	Geoffroy’s bat	*Myotis emarginatus*	Paramyxovirus/Coronavirus	[65]
	Greater mouse-eared bat	*Myotis myotis*	Coronavirus/Herpesvirus/Paramyxovirus	[72,73,80,93,94]
	Whiskered bat	*Myotis mystacinus*	Bunyavirus/Reovirus/Paramyxovirus	[95,96,97,98]
	Natterer’s bat	*Myotis nattereri*	Coronavirus/Herpesvirus	[94,99,100,101]
	Greater noctule bat	*Nyctalus lasiopterus*	Coronavirus	[72]
	Leisler’s bat	*Nyctalus leisleri*	Coronavirus	[63]
	Common noctule	*Nyctalus noctula*	Hantavirus/Coronavirus/Adenovirus	[67,102,103,104]
	Kuhl’s pipistrelle	*Pipistrellus kuhlii*	Rhabdovirus/Coronavirus/Bunyavirus/Reovirus	[79,105,106]
	Nathusius’s pipistrelle	*Pipistrellus nathusii*	Adenovirus/Coronavirus/Reovirus	[83,97,102,107]
	Common pipistrelle	*Pipistrellus pipistrellus*	Adenovirus/Coronavirus/Herpesvirus/Paramyxovirus	[78,94,102,108,109,110]
	Pygmy pipistrelle	*Pipistrellus pygmaeus*	Astrovirus/Coronavirus	[111]
	Brown long-eared bat	*Plecotus auritus*	Coronavirus/Astrovirus/Herpesvirus/Reovirus	[81,94,101,102]
	Parti-coloured bat	*Vespertilio murinus*	Reovirus/Astrovirus	[68,69,112]
Miniopteridae	Schreiber’s bat	*Miniopterus schreibersii*	Lloviu (filovirus)	[23,113]
Molossidae	European free-tailed bat	*Tadarida teniotis*	Mammalian orthoreovirus (reovirus)	[68,78]
Pteropodidae	Egyptian fruit bat	*Rousettus aegyptiacus*	Marburg and Ravn (filovirus)	[114,115]
